# Treatment of Osteochondral Lesions of the Talus in the Skeletally Immature Population: A Systematic Review

**DOI:** 10.1097/BPO.0000000000002175

**Published:** 2022-05-20

**Authors:** Jari Dahmen, Jason A.H. Steman, Tristan M.F. Buck, Peter A.A. Struijs, Sjoerd A.S. Stufkens, Christiaan J.A. van Bergen, Gino M.M.J. Kerkhoffs

**Affiliations:** * Department of Orthopedic Surgery, Amsterdam Movement Sciences, Amsterdam UMC, Location AMC, University of Amsterdam; † Academic Center for Evidence-based Sports Medicine (ACES), Amsterdam UMC; ‡ Amsterdam Collaboration for Health and Safety in Sports (ACHSS), International Olympic Committee (IOC) Research Center, Amsterdam UMC, Amsterdam; § Department of Orthopedic Surgery, Amphia Hospital, Breda, The Netherlands

**Keywords:** osteochondral lesions of the talus, children, ankle, cartilage

## Abstract

**Introduction:**

Skeletally immature osteochondral lesions of the talus (OLTs) are underreported and little is known about the clinical efficacy of different treatment options. The primary aim of the present study was to investigate the clinical efficacy of different conservative and surgical treatment options. The secondary aim was to assess return to sports (RTS) and radiologic outcomes for the different treatment options.

**Methods:**

An electronic literature search was carried out in the databases PubMed, EMBASE, Cochrane, CDSR, CENTRAL, and DARE from January 1996 to September 2021 to identify suitable studies for this review. The authors separately screened the articles for eligibility and conducted the quality assessment using the Methodological Index for Non-Randomized Studies (MINORS). Clinical success rates were calculated per separate study and pooled per treatment strategy. Radiologic outcomes and sports outcomes for the different treatment strategies were assessed.

**Results:**

Twenty studies with a total of 381 lesions were included. The mean MINORS score of the included study was 7.6 (range: 5 to 9). The pooled success rate was 44% [95% confidence interval (CI): 37%-51%] in the conservative group (n=192), 77% (95% CI: 68%-85%) in the bone marrow stimulation (BMS) group (n=97), 95% (95% CI: 78%-99%) in the retrograde drilling (RD) group (n=22), 79% (95% CI: 61%-91%) in the fixation group (n=33) and 67% (95% CI: 35%-88%) in the osteo(chondral) autograft group (n=9). RTS rates were reported in 2 treatment groups: BMS showed an RTS rate of 86% (95% CI: 42%-100%) without specified levels and an RTS rate to preinjury level of 43% (95% CI: 10%-82%). RD showed an RTS rate of 100% (95% CI: 63%-100%) without specified levels, an RTS rate to preinjury level was not given. RTS times were not given for any treatment option. The radiologic success according to magnetic resonance imaging were 29% (95% CI: 16%-47%) (n=31) in the conservative group, 81% (95% CI: 65%-92%) (n=37) in the BMS group, 41% (95% CI: 18%-67%) (n=19) in the RD group, 87% (95% CI: 65%-97%) (n=19) in the fixation group, and were not reported in the osteo(chondral) transplantation group. Radiologic success rates based on computed tomography scans were 62% (95% CI: 32%-86%) (n=13) in the conservative group, 30% (95% CI: 7%-65%) (n=10) in the BMS group, 57% (95% CI: 25%-84%) (n=7) in the RD group, and were not reported for the fixation and the osteo(chondral) transplantation groups.

**Conclusions:**

This study showed that for skeletally immature patients presenting with symptomatic OLTs, conservative treatment is clinically successful in 4 out of 10 children, whereas the different surgical treatment options were found to be successful in 7 to 10 out of 10 children. Specifically, fixation was clinically successful in 8 out of 10 patients and showed radiologically successful outcomes in 9 out of 10 patients, and would therefore be the primary preferred surgical treatment modality. The treatment provided should be tailor-made, considering lesion characteristics and patient and parent preferences.

**Level of Evidence:**

Level IV—systematic review and meta-analysis.

Osteochondral lesions of the talus (OLTs) are pathologic lesions of the talar cartilage and its subchondral bone. These injuries have a high association with inversion injuries and ankle fractures.^1^ Treatment of OLTs both in the adult and skeletally immature population depends on a high number of factors, such as lesion morphology and size, primary or nonprimary nature of the lesion, alignment of the lower extremity, and other important patient characteristics such as magnitude of symptoms during daily activities and a potential wish to return to sports (RTS).^2^ Two recent studies in the form of systematic reviews found that presently there is no superior treatment strategy for primary nor for secondary OLTs.^3^,^4^ Both reviews as well as about 90% of the literature that has been published the past 2 decades, solely include patients above the age of 18 years old which consequently results in a substantial scarcity of high-quality evidence on the clinical efficacy of different conservative and surgical treatment paradigms for the skeletally immature population. In addition, studies that have been including skeletally immature patients are either of small sample size or outdated.^5^–^8^ However, and clinically fundamentally, the natural history and the tendency of healing of OLTs in the skeletally immature population may potentially be totally different in comparison to the adult population with distinctive associated clinical success rates as a result.^9^ Moreover, up to date, no systematic review has been performed on exclusively the skeletally immature population being affected by an OLT.

The primary aim of the present study is therefore to investigate and summarize the clinical efficacy of different conservative and surgical treatment options for OLTs in the skeletally immature population, the outcomes of which are to be applied in daily clinical care as it can provide evidence-based information to patients and their parents to improve the quality of the decision-making process. The secondary aim is to assess radiologic and sports outcomes after these treatment options. It was hypothesized that patients undergoing different treatment options for OLTs will demonstrate good functional outcomes at follow-up with conservative treatment options showing relatively lower clinical success rates than the surgical interventions.

## METHODS

This systematic review was prospectively registered at the PROSPERO register^10^ (CRD42019130947), and the review was performed in accordance and guided by the Preferred Items for Systematic Reviews and Meta-Analyses (PRISMA) guidelines.^11^

### Search Strategy

An electronic literature search was carried out in the databases PubMed, EMBASE, Cochrane, CDSR, CENTRAL and DARE to identify studies published from January 1996 to September 2021. The search strategy for all electronic databases is outlined in Appendix 1 (Supplemental Digital Content 1, http://links.lww.com/BPO/A501).

### Eligibility Criteria and Study Selection

All studies were independently screened by 2 independent reviewers (J.D. and J.A.H.S.). When there was no agreement, assessment by an independent third investigator (G.M.M.J.K.) would be decisive for inclusion or exclusion. All studies describing the clinical outcomes of any treatment of primary and secondary OLTs in children were included in the present study. The exact inclusion and exclusion criteria are presented in Table 1. Specifically, children were defined as those patients who were reported to have open growth plates/physes in the separate studies. In case this was not specifically (enough) reported, we adhered to the definition of female patients being 15 years old or younger and male patients being 16 years old or younger,^12^ meaning that all individual included patients, must be under the abovementioned cutoff ages.

**TABLE 1:** Inclusion and Exclusion Criteria

Inclusion Criteria	Exclusion Criteria
Skeletally immature patients with reported open growth plates or physes	Follow-up <1 y after initiation of treatment
Both primary and nonprimary OLTs	Treatment option inappropriately described
All treatment strategies (conservative and surgical therapies)	<5 patients included
Full-text articles available in English, Dutch, German, French, Spanish, and/or Portuguese	Duplicate publications including an overlap of patients
Level I-IV evidence	Level V studies and animal or cadaveric studies

OLT indicates osteochondral lesion of the talus.

### Quality Assessment

For assessing the methodological quality of the studies in this systematic review, the Methodological Index for Non-Randomized Studies (MINORS) instrument was used (Appendix 2, Supplemental Digital Content 2, http://links.lww.com/BPO/A502).^13^ Each included study was graded on methodological quality by 2 independent reviewers (J.D. and J.A.H.S.). When there was no agreement on points graded per the study, assessment by a third independent investigator (G.M.M.J.K.) would be decisive.

### Data Extraction

By means of a standardized extraction form, data extracted from the studies included in this review consisted of characteristics of the study and patient data. The study characteristics extracted included author, title, type/level of evidence, year of publication, clinical scoring systems used, damage classification, treatment(s) used, and range of follow-up time. Patient data extracted from the studies included number of patients, mean age, sex, number of ankles, location of lesion, and outcomes of the clinical scoring system utilized in the study. When the patient data or study characteristics were not represented in the article, the study was not absorbed in the calculation of the overall average of study characteristics. Preoperative and postoperative clinical outcomes including sports outcomes, were extracted and included mean scores, percentage of patients treated successfully, percentage of patients returning to sports (with or without regard to preinjury levels) and return to sport times. In case of the presence of radiologic scores or outcomes, these were additionally extracted from the studies and subsequently analyzed. Described treatment techniques were examined per the study, after which they were divided into corresponding treatment groups, similar to the articles published by Dahmen et al^3^ and Lambers et al.^4^ Conservative treatment was defined as any treatment not involving any surgical procedures aiming to resolve or reduce complaints caused by the OLT, such as physiotherapy or immobilization.

### Statistical and Data Analysis

Statistical analysis was performed by pooling data of individual participant data within 1 treatment group (where appropriate: ie, quantitative synthesis was utilized if the included studies are sufficiently homogenous in methodological nature).

The primary outcome measure of this study was the clinical success rate per treatment group/strategy. A treatment strategy was defined to be clinically successful when a good or excellent result at follow-up was reported, in combination with an accepted scoring system. An ankle was also considered to be successfully treated when an American Orthopedic Foot and Ankle Score (AOFAS) at or above 80 was reached. When a reoperation after a prior surgical intervention or a primary surgical intervention after conservative treatment was required, the initial specific treatment strategy was deemed unsuccessful. Reoperations in this matter were solely defined as the ones aimed at the OLT itself (ie, re-do BMS after a prior BMS, osteochondral autografting after BMS, etc.), and were not defined as those aiming to resolve symptoms resulting from secondary causes (eg, impingement). When a study reported clinical outcomes by means of multiple scoring systems, the most frequently utilized scoring system among all included studies was used. A simplified pooling method was used to combine data from different studies using corresponding methodologies to provide results within 1 treatment group. Ninety-five percent confidence intervals (CIs) (binomial proportion) for the success rates of each study and the pooled studies were calculated with the Wilson score interval. A comparison of different clinical and radiologic outcomes by means of formal statistical tests with accompanying *P*-values was not deemed methodologically suitable for the present review, as the specific clinical indications for specific therapeutic options were highly different from one another.^3^,^4^

Secondary outcomes were RTS and radiologic outcomes. We distinguished 2 types of RTS: return with level specified, where the patients had to perform in the same sport at the same level as preinjury; and return without a specified level, where the patients could participate in any sport at any level, regardless of their performance before their injury.

Radiologic results were assessed through a calculated success rate. When there were no remaining signs of the lesion, on either computed tomography (CT) scan or a magnetic resonance imaging (MRI) scan, a treatment strategy was deemed radiologically successful. In case of results being reported by means of a radiologic scoring system, individual scores per patient were extracted. An excellent or good result was qualified as being successful. The 2 abovementioned outcomes were combined to calculate a radiologic success rate separately for results after CT and MRI. Ninety-five percent CIs were calculated with the Wilson score interval. Radiologic results regarding osteoarthritic/degenerative changes were pooled and analyzed separately whenever possible.

## RESULTS

### Search Results

**FIGURE 1:**
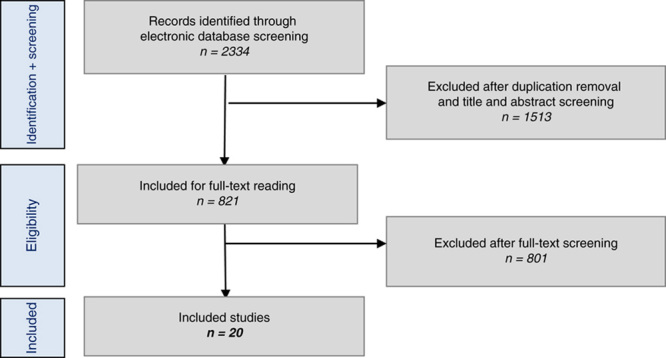
Literature selection algorithms—Preferred Reporting Items for Systematic Reviews and Meta-Analyses (PRISMA).

The literature search using the databases as stated above provided 2334 articles. After duplicate removal and application of eligibility criteria to the titles and abstracts, 821 articles were included for the full-text review. Subsequently, the full-text articles were screened and the inclusion and exclusion criteria were applied. A total of 801 articles had to be excluded after full-text screening. This left 20 suitable studies which were included in the present review. The literature selection algorithm according to PRISMA is shown in Figure 1.^11^

### Characteristics of Included Studies

The 20 included studies yielded a total of 353 patients with a total of 381 OLTs. The average age was 13 years (range: 2 to 16 y), and the percentage of females and males was 44% and 56%, respectively. The AOFAS and the Berndt and Harty classification system were the most frequently used scoring systems used for clinical and radiologic scoring, respectively. Seven studies reported radiologic results after MRI, and 3 studies reported radiologic results after CT. The mean follow-up time was 59 months (range: 16 to 432 mo).

### Methodological Quality

After independent grading and discussion by 2 reviewers (J.D. and J.A.H.S.), a full consensus on methodological quality was reached. All of the 20 included studies were noncomparative retrospective studies. The average MINORS score of the studies was 7.6 (range: 5 to 9) out of a possible 16 points. A full overview of the scores per study is shown in Appendix 3 (Supplemental Digital Content 3, http://links.lww.com/BPO/A503).

### Treatment Strategies

All studies reporting different treatment strategies were pooled into 6 different treatment strategy groups (Appendix 4, Supplemental Digital Content 4, http://links.lww.com/BPO/A504). The combined number of treatment strategies was higher than the total number of included studies, as several studies reported outcomes after different treatment strategies. As all included studies had corresponding methodological natures (all retrospective case series), a simplified pooling method could be performed for studies that reported on the same outcomes after performing the same treatment technique.

An overview of the study characteristics, patient demographics, and outcomes for the different treatment groups is shown in Table 2.

**TABLE 2:** Overview of Study Characteristics, Patient Demographics, and Outcomes

	Conservative Treatment	Bone Marrow Stimulation	Retrograde Drilling	Fixation
No. studies	8 studies^6^–^8^,^14^–^18^	8 studies^6^,^7^,^15^,^19^–^23^	6 studies^9^,^14^,^16^,^24^–^26^	4 studies^7^,^21^,^27^,^28^
Study types	8 retrospective case series	8 retrospective case series	6 retrospective case series	4 retrospective case series
Mean follow-up duration (mo)	16-144	16-67	24-70	40-48
Patient age (range of individual data) (y)	4-16	6-17	8-16	12-16
No. lesions	192	119	106	33
Primary/secondary lesions	192/0	119/0	106/0	33/0
History of trauma	Available for 63 lesions 37 (59%) with a history of trauma	Available for 45 lesions 30 (67%) with a history of trauma	Available for 14 lesions 7 (50%) with a history of trauma	Available for 5 lesions 2 (40%) with a history of trauma
Lesion size (mean)	Available for 37 lesions 131 mm^2^	Available for 21 lesions 131 mm^2^	Available for 8 lesions 90 mm^2^	Available for 19 lesions 164 mm^2^
Lesion location	Available for 95 lesions 67 medial 7 central 21 lateral	Available for 33 lesions 31 medial 0 central 2 lateral	Available for 14 lesions 12 medial 0 central 2 lateral	Available for 5 lesions 3 medial 0 central 2 lateral
Berndt and Harty staging	Available for 190 lesions 21 stage I (11%) 80 stage II (42%) 80 stage III (42%) 9 stage IV (5%)	Available for 119 lesions 12 stage I (10%) 52 stage II (44%) 48 stage III (40%) 7 stage IV (6%)	Available for 78 lesions 11 stage I (14%) 44 stage II (57%) 21 stage III (27%) 2 stage IV (2%)	Available for 27 lesions 2 stage I (7%) 9 stage II (34%) 14 stage III (51%) 2 stage IV (8%)
RTS rates regardless of level (pooled results of individual data)	Not reported	Available for 37 patients (1 study^21^,^23^) 86% (95% CI: 42%-100%)	Available for 8 patients (1 study^24^) 100% (95% CI: 63%-100%)	Not reported
RTS rates to preinjury (pooled results of individual data)	Not reported	Available for 37 patients (1 study^23^) 43% (95% CI: 10%-82%)	Not reported	Not reported
RTS times (pooled results of individual data)	Not reported	Not reported	Not reported	Not reported
Osteoarthritic changes (pooled results of individual data)	Not reported	Available for 21 patients^7^ 0% (95% CI: 0%-16%) with signs of osteoarthritis	Available for 21 patients^26^ 19% (95% CI: 9%-36%) with worsening of osteoarthritis	Available for 14 patients^21^ 21% (95% CI: 5%-51%) with signs of osteoarthritis

CI indicates confidence interval; RTS, return to sports.

### Conservative

The aim of conservative treatment is to facilitate the natural healing potential of the damaged tissue and resolve edema within the joint by means of the avoidance of weight-bearing. Pain and inflammation can be treated using nonsteroidal anti-inflammatory drugs. In addition, intra-articular hyaluronic acid or platelet-rich plasma injections can be given to aim to reduce pain and to increase function.^29^,^30^ Eight studies were identified on conservative management, and the protocol used for conservative treatment was described by 5 out of 8 included studies^6^,^7^,^14^–^16^ and consisted of immobilization for a mean period of 6 weeks (range: 3 to 8 wk), followed by a period of restricted weight-bearing and sports activities, with a mean time of 5 months (range: 4 to 6 mo). Three studies adhered to a cast immobilization protocol for a total of 55 patients, whereas 2 studies adhered to an immobilization protocol without the usage of cast for a total of 113 patients.

A simplified pooling method was performed for all 8 studies reporting clinical outcomes after conservative treatment. This yielded a pooled clinical success rate of 44% (95% CI: 37%-51%) for a total of 192 patients. One study reported separate results for lesions with and without a history of trauma,^6^ yielding a success rate of 26% (95% CI: 12%-49%) for traumatic lesions and 14% (95% CI: 3%-51%) for lesions without a history of trauma, for a total of 19 and 7 lesions, respectively. This study also reported separate results with regard to lesion location, yielding a success rate of 26% (95% CI: 12%-49%) for 19 medial lesions, 0% (95% CI: 0%-66%) for 2 central lesions, and 0% (95% CI: 0%-43%) for 5 lateral lesions. Three studies reported separate results with regard to Berndt and Harty staging,^6^,^7^,^17^ yielding success rates of 63% (95% CI: 39%-82%) for 16 stage I lesions, 42% (95% CI: 29%-55%) for 53 stage II lesions, 23% (95% CI: 12%-39%) for 35 stage III lesions, and 0% (95% CI: 0%-66%) for 2 stage IV lesions.

Four studies reported on conversion to surgery^6^,^7^,^14^,^16^ yielding a conversion to surgery rate of 62% (95% CI: 54%-70%) for a total of 157 patients. Two studies reported radiologic results after using MRI,^16^,^17^ yielding a pooled success rate of 29% (95% CI: 16%-47%) for a total of 31 patients. One study reported on radiologic results after using CT^8^ with a success rate of 62% (95% CI: 32%-86%) for a total of 13 patients.

### Bone Marrow Stimulation (BMS) (Debridement and/or Drilling)

BMS aims at forming new local blood vessels and stimulating fibrocartilaginous tissue. This is done by debriding and additionally microfracturing or antegrade drilling. This allows a blood clot to form and the release of growth factors, resulting in formation of fibrocartilaginous tissue.

A simplified pooling method was performed for 6 studies reporting clinical outcomes after treatment with BMS. This yielded a pooled success rate of 77% (95% CI: 68%-85%) for a total of 97 patients. One study reported separate results for lesions with and without a history of trauma,^6^ yielding a success rate of 100% (95% CI: 61%-100%) for traumatic lesions and 100% (95% CI: 51%-100%) for lesions without a history of trauma, for 6 and 4 lesions, respectively. Two studies also reported separate results with regard to lesion location,^6^,^19^ yielding a success rate of 95% (95% CI: 76%-99%) for 20 medial lesions, these studies did not include any central or lateral lesions. One study also reported separate results with regard to Berndt and Harty staging,^20^ but only included stage II lesions, yielding a success rate of 100% (95% CI: 72%-100%) for 10 lesions.

Two studies reported on reoperation rate,^7^,^21^ yielding a rate of 24% (95% CI: 14%-37%) for a total of 58 patients. One study reported on radiologic results after MRI,^21^ with a success rate of 81% (95% CI: 65%-92%) for a total of 37 patients. One study reported on radiologic results after using CT,^20^ with a success rate of 30% (95% CI: 7%-65%) for a total of 10 patients.

### Retrograde Drilling (RD)

The aim of RD is to revascularize the subchondral bone and induce novel bone formation. RD is a nontransarticular procedure, preventing injury to the articular cartilage. A simplified pooling method was performed for 3 studies reporting clinical outcomes after treatment with RD.^9^,^24^ This yielded a pooled success rate of 95% (95% CI: 78%-99%) for a total of 22 patients. Two studies reported separate results for lesions with and without a history of trauma,^9^,^24^ yielding a success rate of 86% (95% CI: 47%-97%) for traumatic lesions and 100% (95% CI: 65%-100%) for lesions without a history of trauma, for a total of 7 and 7 lesions, respectively. Three studies also reported separate results with regard to lesion location,^9^,^24^ yielding a success rate of 95% (95% CI: 76%-99%) for 20 medial lesions and 100% (95% CI: 34%-100%) for 2 central lesions, these studies did not include any lateral lesions. Two studies reported separate results with regard to Berndt and Harty staging,^9^,^24^,^25^ yielding success rates of 100% (95% CI: 57%-100%) for 5 stage I lesions, 100% (95% CI: 68%-100%) for 8 stage II lesions, and 0% (95% CI: 0%-79%) for 1 stage III lesion, these studies did not include any stage IV lesions. No results on reoperations were given. Two studies reported on radiologic results after using MRI,^9^,^16^ yielding a success rate of 41% (95% CI: 18%-67%) for a total of 19 patients. One study reported on radiologic results after using CT,^25^ with a success rate of 57% (95% CI: 25%-84%) for a total of 7 patients.

### Fixation

This treatment technique can be considered when large osteochondral fragments are apparent in the joint. The loose osteochondral fragment is fixated to the underlying bone, using bioabsorbable pins, screws, Kirschner wires, bone pegs, or fibrin glue.^31^–^33^ A simplified pooling method was performed for all 4 studies, all retrospective case series, reporting clinical outcomes after fixation treatment. This yielded a pooled success rate of 79% (95% CI: 61%-91%) for a total of 33 patients. One study reported separate results for lesions with and without a history of trauma,^27^ yielding a success rate of 100% (95% CI: 34%-100%) for traumatic lesions and 100% (95% CI: 44%-100%) for lesions without a history of trauma, for a total of 2 and 3 lesions, respectively. This study also reported separate results with regard to lesion location, yielding a success rate of 100% (95% CI: 44%-100%) for 3 medial lesions and 100% (95% CI: 34%-100%) for 2 lateral lesions. This study also reported separate results with regard to Berndt and Harty staging, yielding success rates of 100% (95% CI: 44%-100%) for 3 stage II lesions and 100% (95% CI: 34%-100%) for 2 stage III lesions, these studies did not include any stage I or IV lesions. Reoperation rates were given by 2 studies,^7^,^21^ yielding a rate of 22% (95% CI: 7%-44%) for a total of 23 patients. Two studies reported on radiologic results after using MRI,^21^,^27^ yielding a success rate of 87% (95% CI: 65%-97%) for a total of 19 patients.

### Osteo(Chondral) Transplantation

Osteo(chondral) transplantation techniques aim to reproduce the mechanical, structural and biochemical qualities of the talus by restoring the cartilage and subchondral structures using either the body’s own or foreign tissue. Multiple techniques for osteo(chondral) transplantation are being used, such as mosaicplasty, osteochondral autograft or allograft transfer systems (OATS), cancellous bone grafting and osteoperiosteal cylinder grafting. This group included 1 study,^5^ a retrospective case series reporting results for different surgical treatment options, with 9 lesions treated by osteo(chondral) Transplantation. Mean follow-up was not reported separately for the osteo(chondral) transplantation group. Patient age ranged from 9 to 16 in total, but was not reported separately for the osteo(chondral) transplantation group. All lesions were primary lesions. No separate data was reported on history of trauma, lesion size, lesion location or Berndt and Harty staging. The clinical success rate percentage of the included study was 67% (95% CI: 35%-88%), no separate results for lesions with or without history of trauma, and with regard to lesion location or Bernd and Harty staging were reported. This study did not report reoperation rates, radiologic results or RTS. This study did report on osteoarthritic changes for all 9 patients, with 0% (95% CI: 0%-25%) of patients showing signs of osteoarthritis.

**FIGURE 2:**
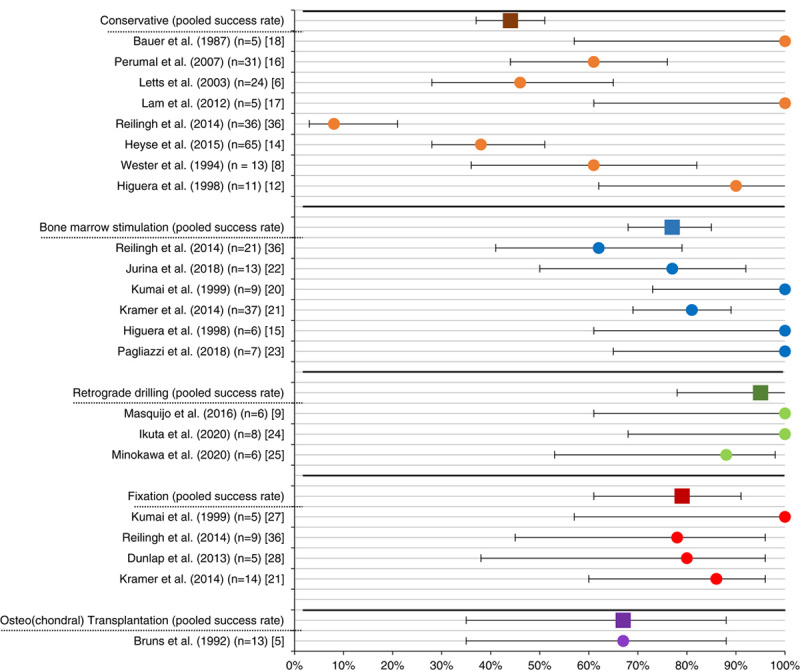
Forest plot of the clinical success rates per separate study and pooled per treatment option.

A forest plot of our primary outcome measure, the pooled clinical success rates per treatment option, is shown in Figure 2.

## DISCUSSION

To the best of our knowledge, this is the first systematic review summarizing the outcomes of conservative and surgical management options for skeletally immature patients with an OLT.

The most important finding is that this study showed that conservative treatment is clinically successful in 4 out of 10 children, whereas the different surgical treatment options are successful in 7 to 10 out of 10 children. This study will serve as an important augmentation to the evidence on treatment of skeletally immature osteochondral lesions to improve shared decision-making between children, their parents and caregivers.

Conservative management was the most reported intervention for treatment in the skeletally immature population, which is in contrast with the findings in the adult population of Dahmen et al,^3^ who reported BMS as the most used intervention. This difference can be explained by the fact that children have a greater healing potential compared with adults.

This healing potential in the skeletally immature population can be supported by the radiologic success rate which ranged between 29% and 62%. This is in contrast with the findings by Seo et al,^34^ who reported a rate of 6% in the adult population. In addition, younger patients were included in the group who underwent conservative management, compared with those in the groups that underwent surgical treatment. It is possible that patients who benefit from conservative management are younger and have other lesion characteristics than patients who needed surgery. The pooled success rate of 44% is substantially higher compared with the adult population. However, it is debatable whether this is high enough, considering the burden of the lengthy conservative treatment protocol, especially for the skeletally immature population.

When focusing on clinical outcomes in the surgical treatment groups, the pooled success rate varied between 67% and 100%. RD reported the highest success rate, followed by fixation and BMS. A substantial drawback of BMS, however, is the formation of collagen type 1/fibrocartilage. This tissue has poorer weight-bearing properties when compared with the natural hyaline cartilage of the talus, and this could result in degeneration of the fibrocartilage tissue and lead to osteoarthritic changes over time.^35^ The found success rates are similar to those found in adults in the review by Dahmen et al.^3^ Although these rates seem satisfactory, better results were expected due to the greater healing potential in the skeletally immature population. The similar rates that were found could also be due to that a high number of the lesions are being treated in a conservative manner for the skeletally immature population, as such concentrating higher or more severe levels of OLT pathologies into the operative cohorts that have been published in the literature. When trying to differentiate the clinical outcomes for traumatic and nontraumatic lesions, it was found that data on trauma history was underreported in the included studies. Therefore, no conclusions on differences in clinical outcomes between traumatic and nontraumatic lesions could be drawn.

The highest radiologic success rate was 87%, which was observed in the fixation group. This high rate of healing can be explained by the indication for fixation, which is a large osteochondral fragment without signs of osteoarthritis grade ≥2.^2^ Due to the fixation of the fragment, the natural congruency of the talus is maintained, and the hyaline cartilage is preserved which possibly contributes to the greater radiologic success. Moreover, superior healing of the subchondral bone after the fixation technique is reported.^36^ Fixation may have an even higher healing rate in traumatic lesions compared with nontraumatic lesions, taking into account the differences in their developing mechanisms. We could, however, not formally test this hypothesis in the present study due to the limited data on this particular outcome of interest. This should therefore be suggested as a future issue of research focus.

Osteoarthritis varied between 0% and 21% in all treatment groups. This range is in contrast with the findings of Edmonds et al^26^ who found an osteoarthritis rate of ∼25%. However, the follow-up time in the study of Edmonds et al^26^ was 192 months, which is near the upper limit of the follow-up range in the included studies of this systematic review. Due to this relatively long follow-up, differences in OA rates can be explained by the fact that OA is more likely to occur in studies with a longer follow-up.

The clinical relevance of the present systematic review is that it provides a clear and concise overview of clinical and radiologic results which can influence the choice of treatment. Information on outcomes in each treatment group could help to provide evidence-based information to patients and their caretakers, which can be used to give patients an indication on expected outcomes when undergoing treatment. This can contribute to the clinical and shared decision-making process. However, various patient-specific and lesion-specific characteristics have to be considered in discussing the optimal treatment for the individual patient.

Aiming to discuss clinical recommendations in the light of the outcomes of the present review and the recent international consensus statements, one can state that the outcomes from our review are supported by the statements generated from the international consensus meeting held in 2019 by the International Consensus Group on Cartilage Repair. A (start with a) conservative treatment protocol is substantiated by a success rate of 44% as summarized in the present review and this was also substantiated by the International Consensus Group on Cartilage Repair of the ankle stating with a strong consensus that all symptomatic pediatric ankle cartilage lesions may initially be managed nonoperatively.^37^ Considering the suggested protocol to perform BMS on patients with small osteochondral lesions to the talus after failed conservative treatment, it can be observed that when summarizing the literature, a clinical success rate of 77% was found which is being supported by international experts in the field stating that these surgical treatment options are to be considered for symptomatic pediatric osteochondral lesions to the ankle after failure of conservative treatment. Specifically, for OLTs that have a fragmentous morphology (intra-articular osteochondral fragment), it is the aim to reach consolidation/union of this fragment, analogous to the goal of arthroscopic or open fixation of the fragment. Therefore, (arthroscopic) fixation strategies being for both acute displaced fragmentous lesions (osteochondral fractures) of the talar dome as well as chronic fragmentous osteochondral lesions which failed conservative management are supported by the outcomes of the present analysis as well, with an overall success rate of 79% for this treatment strategy. A unanimous agreement on this particular suggestion was also stated in the international consensus meeting, thereby substantiating our suggesting to a greater extent.^37^ Moreover, other surgical procedures are possible after failed fixation, for example, arthroscopic BMS or autografting of the OLT. For lesions that are large and/or cystic in nature after failed conservative management, after failed fixation or after failed BMS that can be treated with an OATS procedure or the newly introduced Talar OsteoPeriostic grafting from the Iliac Crest (TOPIC), a success rate of 67% was found in the present analysis.

### Strengths and Limitations

This study has several strengths. First, the methodological quality of this review, containing a standardized, double-controlled method, was used for the selection of the studies. Second, the data synthesis, in the form of a simplified pooling method, provided a fundament to draw conclusions based on the results of this study. Third, both radiologic and clinical results were included in the analysis.

The review also has limitations. The mean MINORS score of 7.6 reveals that the included studies were of limited quality. All included studies were retrospective case series with a wide range in follow-up time, mostly including short to mid-term follow-ups, thereby causing a considerable risk of bias. For studies not reporting on physeal status, a cutoff age of 15 years for girls and 16 years for boys was used, this introduces a risk of bias, as for some patients physeal arrest may have already occurred at these ages and potentially, some skeletally mature patients may have been included Moreover, it must be noted that due to the low number of included patients per study a well-powered novel prospective study could potentially adjust the findings of our present review. However, our study does give clinicians the best current evidence. The most reported clinical outcome in the included studies was the AOFAS. This score seems to be inappropriate for this population as it has never been validated in the skeletally immature population. Therefore, there is a significant chance on bias as overestimation or underestimation of the results are likely to occur. Conclusively, the results of the present study including the summative analysis on the clinical, radiologic as well as return to sport outcomes should be interpreted with caution.

## CONCLUSIONS

This study showed that for skeletally immature patients presenting with symptomatic OLTs, conservative treatment is clinically successful in 4 out of 10 children, whereas the different surgical treatment options were assessed to be successful in 7 to 10 out of 10 children. Specifically, fixation was clinically successful in 8 out of 10 patients and showed radiologically successful outcomes in 9 out of 10 patients, and would therefore be the primary preferred surgical treatment modality. The treatment provided should be tailor-made, considering lesion characteristics and patient and parent preferences.

## Supplementary Material

SUPPLEMENTARY MATERIAL

Supplemental Digital Content is available for this article. Direct URL citations appear in the printed text and are provided in the HTML and PDF versions of this article on the journal's website, www.pedorthopaedics.com.
