# Supplementary material for: Treatment of Osteochondral Lesions of the Talus in the Skeletally Immature Population: A Systematic Review
Source: J Pediatr Orthop. 2022 May 20;42(8):e852–60. doi: 10.1097/BPO.0000000000002175 (PMC9351694; doi:10.1097/BPO.0000000000002175)
Supplement: SUPPLEMENTARY MATERIAL [file bpo-42-e852-s001.docx]

**Appendix 1.** Electronic search strategy used in this review

| **#** | **Searches** | **Results** |
| --- | --- | --- |
| 1 | "Osteochondritis Dissecans"[Mesh] | Total number of results  2026 hits |
| 2 | osteochondritis dissecans[tiab] OR osteochondrosis dissecans[tiab] OR osteochondrolysis[tiab] OR OCD[tiab] OR OLT[tiab] |  |
| 3 | (osteochondral[tiab] OR chondral[tiab] OR transchondral[tiab] OR cartilage*[tiab]) AND (defect*[tiab] OR lesion*[tiab]) |  |
| 4 | #1 OR #2 OR #3 |  |
| 5 | "Talus"[Mesh] |  |
| 6 | talus[tiab] OR talar*[tiab] OR ankle[tiab] |  |
| 7 | #5 OR #6 |  |
| 8 | #4 AND #7 |  |
