# Supplementary material for: Treatment of Osteochondral Lesions of the Talus in the Skeletally Immature Population: A Systematic Review
Source: J Pediatr Orthop. 2022 May 20;42(8):e852–60. doi: 10.1097/BPO.0000000000002175 (PMC9351694; doi:10.1097/BPO.0000000000002175)
Supplement: SUPPLEMENTARY MATERIAL [file bpo-42-e852-s003.docx]

**Appendix 3**. MINORS scores per study

| **Quality assessment non-randomized trials** | Kumai | Kumai | Letts | Perumal | Lam | Reilingh | Kramer | Masquijo | Heyse | Jurina | Pagliazzi | Bruns | Higuera | Edmonds | Ikuta | Carlson | Bauer | Wester | Dunlap | Minokawa |
| --- | --- | --- | --- | --- | --- | --- | --- | --- | --- | --- | --- | --- | --- | --- | --- | --- | --- | --- | --- | --- |
| **Treatment group** | BMS | Fix | Cons / | Cons / | Cons | Cons / | BMS / | RD | Cons / | BMS | BMS | O(C)T | Cons / | RD | RD | BMS | Cons | Cons | Fix | RD |
|  |  |  | BMS | RD |  | BMS / Fix | Fix |  | RD |  |  |  | BMS |  |  |  |  |  |  |  |
| A clearly stated aim | 1 | 2 | 1 | 2 | 1 | 2 | 2 | 1 | 2 | 1 | 1 | 2 | 0 | 2 | 2 | 1 | 2 | 0 | 1 | 1 |
| Inclusion of consecutive patients | 0 | 2 | 2 | 2 | 2 | 2 | 2 | 2 | 2 | 2 | 2 | 0 | 2 | 2 | 0 | 2 | 2 | 2 | 2 | 2 |
| Prospective data collection | 0 | 0 | 0 | 0 | 0 | 0 | 0 | 0 | 0 | 0 | 0 | 0 | 0 | 0 | 0 | 0 | 0 | 0 | 0 | 0 |
| Endpoints appropriate to the aim of the study | 2 | 2 | 2 | 2 | 2 | 2 | 2 | 2 | 2 | 2 | 2 | 2 | 2 | 2 | 2 | 2 | 2 | 2 | 2 | 2 |
| Unbiased assessment of the study endpoint | 0 | 0 | 0 | 0 | 0 | 0 | 0 | 0 | 0 | 0 | 0 | 0 | 0 | 0 | 0 | 0 | 0 | 0 | 0 | 0 |
| A follow-up period appropriate to the aims of study | 2 | 2 | 0 | 1 | 1 | 2 | 2 | 2 | 2 | 2 | 2 | 1 | 2 | 2 | 2 | 2 | 2 | 2 | 2 | 2 |
| Less than 5% loss to follow-up | 1 | 0 | 1 | 1 | 1 | 1 | 1 | 1 | 1 | 1 | 1 | 1 | 1 | 1 | 1 | 1 | 1 | 1 | 1 | 1 |
| Prospective calculation of the sample size | 0 | 0 | 0 | 0 | 0 | 0 | 0 | 0 | 0 | 0 | 0 | 0 | 0 | 0 | 0 | 0 | 0 | 0 | 0 | 0 |
| An adequate control group |  |  |  |  |  |  |  |  |  |  |  |  |  |  |  |  |  |  |  |  |
| Contemporary groups |  |  |  |  |  |  |  |  |  |  |  |  |  |  |  |  |  |  |  |  |
| Baseline equivalence of groups |  |  |  |  |  |  |  |  |  |  |  |  |  |  |  |  |  |  |  |  |
| Adequate statistical analyses |  |  |  |  |  |  |  |  |  |  |  |  |  |  |  |  |  |  |  |  |
| **Total score** | 6 | 8 | 6 | 8 | 7 | 9 | 9 | 8 | 9 | 8 | 8 | 6 | 7 | 9 | 7 | 8 | 9 | 7 | 8 | 8 |
