# Supplementary material for: Treatment of Osteochondral Lesions of the Talus in the Skeletally Immature Population: A Systematic Review
Source: J Pediatr Orthop. 2022 May 20;42(8):e852–60. doi: 10.1097/BPO.0000000000002175 (PMC9351694; doi:10.1097/BPO.0000000000002175)
Supplement: SUPPLEMENTARY MATERIAL [file bpo-42-e852-s004.docx]

**Appendix 4:** Included Treatment Groups

| 20 included studies: 27 treatment strategies |
| --- |
| **8 Conservative** |
| - 8 conservative |
| **8 Bone Marrow stimulation** |
| - 7 BMS without additional therapies |
| - 1 BMS with additional BMAC |
| **6 Retrograde drilling** |
| - 6 retrograde drilling with cancellous bone grafting |
| **4 Fixation** |
| - 4 Drilling + fixation |
| **1 Osteo(chondral) transplantation** |
| - 1 Autologous bone transplantation |
